# Preoperative stereotactic radiosurgery for brain metastases: the STEP study protocol for a multicentre, prospective, phase-II trial

**DOI:** 10.1186/s12885-021-08602-0

**Published:** 2021-07-28

**Authors:** Angeline Ginzac, Guillaume Dupic, Lucie Brun, Ioana Molnar, Mélanie Casile, Xavier Durando, Pierre Verrelle, Jean-Jacques Lemaire, Toufic Khalil, Julian Biau

**Affiliations:** 1 INSERM U1240 IMoST, University of Clermont Auvergne, Clermont-Ferrand, France; 2 Centre d’Investigation Clinique UMR 501, Clermont-Ferrand, France; 3 Department of Clinical Research, Délégation Recherche Clinique et Innovation, Centre Jean PERRIN, Clermont-Ferrand, France; 4 Radiation Department, Centre Jean PERRIN, Clermont-Ferrand, France; 5 Oncology Department, Centre Jean PERRIN, Clermont-Ferrand, France; 6 University of Clermont Auvergne, UFR Médecine, Clermont-Ferrand, France; 7 Department of Radiation Oncology, Institut Curie, Paris, France; 8 Department of neurosurgery, Clermont-Ferrand University Hospital, Clermont-Ferrand, France

**Keywords:** Brain metastases, Preoperative stereotactic radiotherapy, Local control, Radio-necrosis, Overall survival

## Abstract

**Background:**

Surgery is an important therapeutic option for brain metastases. Currently, postoperative stereotactic radiosurgery (SRT) leads to 6-month and 1-year local control estimated at 70 and 62% respectively. However, there is an increased risk of radio-necrosis and leptomeningeal relapse. Preoperative SRT might be an alternative, providing local control remains at least equivalent. It is an innovative concept that could enable the stereotactic benefits to be retained with advantages over post-operative SRT.

**Methods:**

STEP has been designed as a national, multicentre, open-label, prospective, non-randomized, phase-II trial. Seventeen patients are expected to be recruited in the study from 7 sites and they will be followed for 12 months. Patients with more than 4 distinct brain metastases, including one with a surgical indication, and an indication for SRT and surgery, are eligible for enrolment. The primary objective of the trial is to assess 6-month local control after preoperative SRT. The secondary objectives include the assessment of local control, radio-necrosis, overall survival, toxicities, leptomeningeal relapse, distant control, cognitive function, and quality of life. The experimental design is based on a Flemming plan.

**Discussion:**

There is very little data available in the literature on preoperative SRT: there have only been 3 American single or two-centre retrospective studies. STEP is the first prospective trial on preoperative SRT in Europe. Compared to postoperative stereotactic radiotherapy, preoperative stereotactic radiotherapy will enable reduction in the irradiated volume, leptomeningeal relapse and the total duration of the combined treatment (from 4 to 6 weeks to a few days).

**Trial registration number:**

Clinicaltrials.gov: NCT04503772, registered on August 07, 2020. Identifier with the French National Agency for the Safety of Medicines and Health Products (ANSM): N°ID RCB 2020-A00403–36, registered in February 2020. Protocol: version 4, 07 December 2020.

## Background

The incidence of brain metastases for patients with cancer varies from 9 to 30% [1–3]. However, it is higher for patients whose primary cancer is lung cancer, breast cancer or melanoma [4, 5]. Brain metastases are associated with poorer prognosis and cause severe side effects, such as the deterioration of cognitive functions, with a negative impact on quality-of-life [6].

Surgery is an important treatment option for brain metastases. However, the two-year local relapse rate is high (46 to 59%) for patients treated only with surgery [7, 8].

Historically, the gold standard postoperative treatment was whole brain radiation therapy (WBRT) leading to a decrease in the number of local relapses, ranging from 10 to 27%, and in distant relapses [7, 8]. However, WBRT has a significant negative impact on cognitive functions and quality-of-life [9, 10]. Postoperative stereotactic radiotherapy (SRT) has thus been developed to limit these side effects without negative impact on overall patient survival, and it has now become the new standard [8, 11].

To date, four prospective studies on postoperative SRT have been published and have shown an estimated local control rate of 70% at 6 months and 62% at one year, with an increased risk of radio-necrosis (nearly 18% at one year) and leptomeningeal relapse (nearly 17% at one year) [9, 12–14]. A phase-III randomized study conducted on 194 patients compared WBRT to postoperative SRT [9]. With a median follow-up of 11 months, the authors showed that the survival time without cognitive deterioration was longer for patients treated with postoperative SRT than for those treated with WBRT (respectively, a median of 3.7 months [CI95 3,45-5,06] vs. 3.0 months [CI95 2,85-3,25]; HR 0,47 [CI95 0,35-0,63]; *p* < 0.0001). Furthermore, six-month cognitive deterioration was significantly less frequent for patients treated with postoperative SRT (− 33.6% [CI95–45,3-21,8]; *p* < 0.00031). No differences were found between the groups in terms of overall survival (HR 1,07; [CI95 0,76-1,50]; *p* = 0.7). However, postoperative SRT has several drawbacks: difficulties in delineating the target volume, a high volume of irradiation, a high rate of leptomeningeal recurrence and long overall treatment time (3–6 weeks between surgery and SRT). The alternative to postoperative SRT could be preoperative SRT, providing local control remains at least equivalent. This is an innovative concept that could enable the benefits of SRT to be retained with fewer drawbacks than with postoperative SRT.

To date, three retrospective series of preoperative SRT with a limited number of patients have been published and seem to confirm these advantages [15–17]. The median dose prescribed was 14 Gy (1 fraction) on isodose 80% without any margin around the metastasis. Surgery was most often carried out within 24–48 h following SRT.

A retrospective study conducted by Asher et al. on 47 patients treated with preoperative SRT showed 6-month and 12-month survival rates of 77,8 and 60% respectively [15]. The six-month local control rate was 97,8%, falling to 85,6% and 71,8% at 12 and 24 months respectively. With 12 months’ hindsight, no radio-necrosis or leptomeningeal relapse was found in the cohort.

A retrospective study compared preoperative and postoperative SRT on a cohort of 180 patients [18]. No difference was found in terms of overall survival (*p* = 0,1), local relapse (p = 0,24) or distant relapse (p = 0,75). However, postoperative SRT was associated with 2-year leptomingeal relapse and radio-necrosis rates were higher than those found for preoperative SRT (16,6% vs. 3.2%; *p* = 0.01 and 16.4% vs. 4,9%; p = 0,01 respectively). The authors underlined the need for prospective studies assessing preoperative SRT.

To date, no prospective study on preoperative SRT has been published and none is currently being conducted in Europe. Our phase-II trial aims to evaluate preoperative SRT in the treatment of patients with brain metastases with the hypothesis that the local control provided by preoperative SRT will be at least equivalent to that of postoperative SRT, but with a better safety profile.

## Methods/design

### Study design

STEP has been designed as a national, multicentre, open-label, prospective, non-randomized, phase-II trial to evaluate the efficacy and toxicity of preoperative SRT for patients with brain metastases.

The experimental plan will be run using Fleming’s single-stage design without interim evaluation.

This study has been registered on Clinicaltrials.gov (NCT04503772). Seventy patients are expected to be enrolled. The study was started in February 2021 with an 18-month enrolment period and an estimated completion date by July 2023.

### Coordination and participating institutions

The Centre Jean PERRIN is the sponsor and is responsible for coordination, trial management, data management and trial monitoring.

This multicentre study is currently being conducted in 7 sites in France. The list of the study sites is available on https://clinicaltrials.gov/ct2/show/NCT04503772.

### Study objectives and endpoints

#### Primary objective

##### The primary objective of the STEP study is to assess 6-month local control after preoperative SRT

At the M6 follow-up visit (i.e. 6 months after preoperative SRT), local control will be evaluated on cerebral MRI. Local relapse is defined as the emergence or progression of nodular contrast within the resection cavity according to the RANO-BM criteria.

#### Secondary objectives

##### Assessment of local control at 1 year

At the M12 follow-up visit (i.e. 12 months after preoperative SRT), local control will be evaluated on cerebral MRI.

##### Assessment of the radio-necrosis incidence at 1 year

Radio-necrosis is histologically defined post-operatively, either according to the anatomo-pathological report, or in the absence of salvage surgery, by the appearance of or increase in gadolinium contrast on T1 MRI sequences, associated with an increase in the cerebral blood volume (CBV) ratio (brain blood volume of the tumour / brain blood volume of the non-tumoural white matter) of under 2 on perfusion MRI sequences and/or a standard uptake volume (SUV max) of less than 1.59 on PET scanner at F-DOPA. It will be evaluated at the M12 follow-up visit on cerebral MRI.

##### Overall survival

At each study time-point, patient vital status will be collected. Overall survival is defined as the time between the beginning of preoperative SRT and the date of death from any cause.

##### Acute and delayed toxicities

At each study time-point, except baseline, toxicities will be collected and graded according to NCI CTCAE v5.0. Acute toxicities are those that appear within 3 months after preoperative SRT and delayed toxicities are those that appear more than 3 months after preoperative SRT. In our study, any toxicity ≥4 according to NCI CTCAE v5.0 will be considered as a serious adverse event and will be reported immediately by the investigator to the sponsor according to the local regulations.

##### Assessment of leptomeningeal relapses and assessment of cerebral distant control

This will be assessed using MRI and a clinical examination at each study time-point during follow-up (M3, M6, M9, M12).

##### Assessment of cognitive function and quality of life

At five of the seven study time-points (i.e. baseline, M3, M6, M9 and M12) patients will be asked to complete two questionnaires assessing cognitive functioning and quality of life. These are the Mini Mental State Examination and the EORTC Quality-of-Life questionnaire Core-30.

##### Determination of local control and predictive factors for complications

This will be conducted on the one hand on the basis of morphological criteria such as the doses received, the volume, etc. and on the other on the basis of medical history, concomitant treatments, etc.

##### Determination of survival prognosis factors

(according to patient and tumour characteristics.)

#### Biological analysis

A FFPE block of the brain metastasis will be collected for each patient, unless they oppose it on the informed consent form. This block will be stored by the study sponsor and will be used to conduct translational research.

#### Participant eligibility

The inclusion and non-inclusion criteria are presented in Table 1. Patients will be eligible for the study if they have no more than 4 distinct brain metastases, including one with a surgical indication, an indication for SRT and surgery, and no contraindication for MRI. They will be ineligible if they have metastases from sarcoma or small-cell lung cancer, if they have a documented leptomeningeal disease, or if their survival is estimated under 6 months according to the DS GPA (diagnosis-specific graded prognostic assessment).

**Table 1:** Selection criteria

Inclusion criteria	Non-inclusion criteria
≤ 4 distinct brain metastases, one with surgical indication	Lymphoma, leukaemia, multiple myeloma, germinal tumours or cerebral primary cancer
Diagnosis of histologically proven breast, digestive, non-small cells lung cancer, kidney or melanoma	Metastases from small-cells lung cancer or sarcoma
≤ 5 cm larger diameter	Mass effect with deflection ≥5 mm from median line or hydrocephaly or compression 4th ventricle, patient neurologically unstable, need for emergency decompressive surgery
Karnofsky performance status ≥70	> 4 brain metastases
No contraindication for MRI	Contraindication to anaesthesia, MRI or gadolinium injection
Possibility for the patient to be treat with both surgery and stereotactic radiotherapy	Proximity of the tumour with organs at risk which do not allow the prescribed dose to be reached in the envelope
≥ 18 years old	Pregnant or breastfeeding woman
Estimated overall survival ≥6 months according to DS GPA	Anti VEGF within 6 weeks before treatment
Written inform consent signed	Documented leptomeningeal injury
Affiliation to the French social security system	History of irradiation of the encephalon in toto
For women of childbearing age including those on LH-RH agonists for ovarian suppression: inclusion negative serum pregnancy test (≤ 7 days prior to the start of preoperative RSH).	History of stereotactic radiotherapy on metastasis to be operated on
	Non-candidate patient for surgery
	Surgical delay > 3 days compared to stereotactic radiotherapy
	Estimated survival < 6 months by DS GPA
	Patient under guardianship or curatorship
	Psychological disorder (cognitive disorders, mental alertness, etc.) or social (deprivation of liberty by judicial or administrative decision) or geographical reasons that may compromise medical monitoring of the trial or compliance with treatment
	Woman of childbearing age without effective contraception
	Patient participating in another intervention study within 4 weeks prior to inclusion

#### Intervention: preoperative stereotactic radiosurgery

##### Patient positioning and data acquisition

All patients will be irradiated in supine position. Immobilization devices such as stereotactic customized masks will be used to ensure the accuracy and reproducibility of patient positioning during SRT. For all patients, a dosimetric MRI will be required. The dosimetric MRI sequences of interest will be matched with the planning Computed Tomography (Planning-CT). The maximum time lapse between dosimetric MRI and the first fraction of SRT is 7 days.

##### Volume definition

*Delineation of the target volumes*

The gross tumour volume (GTV) is defined as the contrast-enhanced tumour post-gadolinium contrast-enhanced T1-weighted MRI sequences. The GTV will then be extended symmetrically by 2 mm in all dimensions to create the planning target volume (PTV).

*Delineation of organs at risk*

The following organs at risk will be delineated: healthy brain (corresponding to cranial cavity-GTV), eyeballs, optic chiasm, cochlea, lenses, pituitary gland, hippocampus, optic nerves, brain stem and spinal cord.

*Dose prescription and overall treatment duration*

23.1Gy will be prescribed on the 70% isodose line to encompass at least 99% of the PTV, corresponding to a 30Gy dose around the GTV. The total dose will be delivered in 3 fractions, every other day.

The doses delivered to target volumes and organs at risk are presented in Table 2 [19, 20].

**Table 2:** Dosimetry criteria

TARGET VOLUMES	
PTV (GTV + 2 mm)	V23,1Gy ≥ 99%
	Dmax ≤35Gy
GTV	D98% ≥ 29Gy
**ORGANS AT RISK**	
Brain stem	Dmax < 23,1Gy V18Gy < 0,5 cc
Optic chiasm	Dmax< 17,4Gy
Optic nerves	Dmax<15Gy
Spinal cord	Dmax<15Gy
Healthy brain (cranial cavity – GTV)	V23,1Gy < 7 cc
	V18Gy < 20 cc

*Irradiation technique*

All treatment will be performed on LINAC, 6 MV X-ray photons, up to 1400 UM/min. The SRT mode is left open to the participating institutions depending on their equipment.

Doses delivered to target volumes (Dmin, 98, 2%, max GTV, Dmin, 98, 2%, max PTV, % PTV coverage by isodose 70%), to organs at risk (Dmax and D2% for at-risk organs and VxGy for healthy brain) and the quality indicators (Paddick’s conformity index and gradient index) will be recorded.

*Treatment verification and accuracy*

An online review of the optimal patient repositioning system will be systematically performed before each fraction according to each centre’s policy and equipment. Any necessary offset correction will be applied.

*Treatment interruptions / changes*

No changes (major deviations) will be permitted with regard to the target volume selection and delineation, the radiation dose prescriptions or the overall treatment duration. Local investigators will carefully follow their patients during treatment and take all adequate measures to avoid any interruption and/or change in the total dose. It is, however, the local investigator’s responsibility to interrupt treatment delivery if deemed appropriate in the patient’s best interest. Any such interruptions will be recorded in the electronic case report form (eCRF). In case of machine breakdown or a non-working day, all due measures will be taken to avoid prolonging the overall treatment duration.

*Time lapse between SRT and surgery*

Complete removal of the metastasis will be carried out by the neurosurgeon within ≤3 days from the end of the SRT. Postoperative imagery will be performed within 48 h following surgery.

### Study procedures and participant timeline

An overview of the study assessments and procedures is presented in Table 3.

**Table 3:** Data collection schedule

				Follow-up			
	**Inclusion**	**Preop SRS**	**Surgery**	M3	M6	M9	M12
Consent	**✓**						
Medical and surgical history	**✓**						
History of the disease	**✓**						
DS GPA classification	**✓**						
Serum Pregnancy Test (for woman of childbearing age)	**✓**						
Clinical examination	**✓**	**✓**		**✓**	**✓**	**✓**	**✓**
Previous and concomitant treatment	**✓**	**✓**		**✓**	**✓**	**✓**	**✓**
Toxicity Evaluation (NCI CTCAE v5.0)		**✓**	**✓**	**✓**	**✓**	**✓**	**✓**
MMSE and EORTC QLQ-C30 questionnaires	**✓**			**✓**	**✓**	**✓**	**✓**
Surgery information (date, quality of surgery and post-operative follow-up)			**✓**				
Dosimetric brain MRI	**✓**						
Dosimetric CT scan	**✓**						
Tumor sample (FFPE block)	**✓**						
Dose delivered to target volumes and organs at risk		**✓**					
Paddick conformity Index and Gradient Index		**✓**					
Brain MRI			**✓**^a^	**✓**	**✓**	**✓**	**✓**

^a^ within 48 h postoperatively (scanner accepted if MRI is not available)

**Fig. 1:**
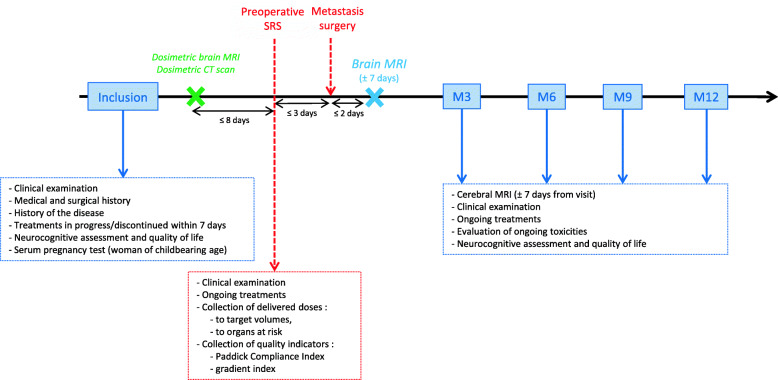
Scheme of the STEP study

Seven consultations are planned for each enrolled patient: inclusion, preoperative SRT, brain metastasis surgery within the 3 following days, and follow-up every three months (M3, M6, M9, M12). The study layout is presented in Fig. 1.

### Statistical analysis

#### Sample size

For sample size calculation, two elements have been taken into account: the primary objective of efficacy at 6 months and the secondary endpoint of 1-year radio-necrosis toxicity.

The efficacy of pre-operative SRT is expected to be at least equivalent to postoperative SRT, estimated at 70%. The experimental plan of the study is Fleming’s one-stage design with a sufficient efficacy threshold set at 86% (higher than rates observed in retrospective studies) and an insufficient efficacy threshold set at 70%. In order to control the (one-sided) type I error rate α at 5% and type II error rate β at 10%, the minimum number of patients to be enrolled should be 57.

For the incidence of radio-necrosis at 1 year, to conclude to a decrease compared to that induced in the case of postoperative SRT, a percentage lower than 5% (as observed in the retrospective series) will be accepted and we decided to reject proportions exceeding 15%. To ensure α < 5% and β < 20%, it is sufficient to include 59 patients. The less restrictive hypothesis in terms of power in this second Fleming’s design was decided on the basis that it concerns a secondary objective.

Thus, by including 70 patients, it is possible to compensate for a proportion of more than 15% missing data (for whatever reason) and hence to ensure adequate power of the study in terms of main objective and first secondary objective (the evaluation of the incidence of radio-necrosis).

The treatment will be considered efficacious (and therefore the trial positive) if at least 56/70 of the patients achieve local control. In this case, the estimate of the probability of success will be 80% (90% confidence interval of 71.3–86.9%, Jeffreys method), at least. The treatment will be considered to induce an acceptable level of radio-necrosis if no more than 5/70 patients have this toxicity, and, in this case, the estimate of the probability of toxicity will be 7% (90% confidence interval 3.3–13.6%, Jeffreys method), at least.

### Data analysis

#### Primary analysis

The main analysis consists in calculating the local control rate at 6 months, with its 90% confidence interval, and the result will be interpreted according to Fleming’s procedure. In the case of non-assessable patients, in order to preserve power and control the alpha risk, the strategy used will be to estimate the local control rate using the Kaplan-Meier method (and to compare it to the rejection rate adjusted on the ratio between the number of assessable patients and the number of patients included).

#### Secondary analyses

The objectives involving descriptive analyses will call on conventional methods: an evaluation of different toxicities at different times using percentages and confidence intervals. The analysis of survival data (evaluation of local control, distant control, and overall survival) will be carried out using the Kaplan-Meier method (estimation of survival rates at different times with confidence intervals based on cumulative hazard), and Cox proportional hazards model (uni- and multivariate regression, possibly LASSO-penalized) to search for predictive factors. To investigate predictive factors for complications, a univariate analysis will be performed first, followed by a multivariate logistic regression (penalized if necessary).

#### Missing data

Missing data will not be replaced (it will be managed by partial elimination). If there is a significant level of missing data, an analysis will be performed to assess its statistical nature and its potential effect on the results. If relevant, an appropriate method of imputation will be envisaged.

### Data management and monitoring

The data collected for the study will be entered on an eCRF (Ennov Clinical) by each centre. The people with access to the data will be the investigators, the clinical research associates, the project leaders and the biostatisticians. They are authorized professionals and are subject to professional secrecy. The investigator will ensure the accuracy, completeness, and consistency of the data recorded (pseudonymized patient data) and of the provision of answers to data queries.

Monitoring reviews will be regularly carried out by a clinical research associate mandated by the sponsor. The objectives will be to ensure the correct conduct of the study in each centre, the recording of data generated in writing, its documentation, recording and reporting, in accordance with the legislative and regulatory provisions in force. Monitoring reports will ensure traceability.

### Independent data monitoring committee (IDMC)

The IDMC will be composed of three experts: a radiotherapist, a neurosurgeon and a methodologist. The objective of the IDMC will be to review all safety data from the study.

Grade-4 toxicity is expected to remain under 5%. The IDMC will meet on the basis of 2 grade ≥ 4 toxicity notifications (NCI CTCAE v5.0).

### Trial status

The STEP trial is currently recruiting. Participant recruitment began in February 2021 and recruitment is expected to end in July 2022. The approved protocol is version 4, 07 December 2020.

## Discussion

The STEP study puts forward several hypotheses on pre-operative SRT compared to post-operative SRT:

- A reduction in the irradiated volume with better visualization of the contours of the metastases, limiting the risks of radio-necrosis;
- A reduction in post-operative dissemination, limiting leptomeningeal recurrences;
- A reduction in the number of patients (≈20%) not treated post-operatively (on account of complications, loss to follow-up, etc.);
- A reduction in the total time of combined treatments: a few days vs. 4 to 6 weeks.

If this study confirms the hypothesis that preoperative SRT reduces the risk of radio-necrosis and local and leptomeningeal recurrence while achieving local control at least equivalent to that of postoperative SRT, the procedure could be a new alternative in the management of patients with brain metastases.

## Data Availability

Not applicable.

## Abbreviations

CBV: Cerebral blood volume
CTV: Clinical target volume
DS-GPA: Diagnosis-specific graded prognostic assessment
eCRF: Electronic case report form
EORTC: European Organisation for Research and Treatment of Cancer
GTV: Gross tumour volume
IDMC: Independent data monitoring committee
MRI: Magnetic resonance imaging
NCI CTCAE: National cancer institute common terminology criteria for adverse events
PET: Positon emission tomography
PTV: Planning target volume
RANO-BM: Response Assessment in Neuro-Oncology Brain Metastases
SRT: Stereotactic radiotherapy
SUV max: Maximum standardized uptake value
WBRT: Whole brain radiotherapy
